# FGF- and SHH-based molecular signals regulate barbel and craniofacial development in catfish

**DOI:** 10.1186/s40851-019-0135-1

**Published:** 2019-06-14

**Authors:** Tatsuya Itoyama, Makiko Fukui, Masahumi Kawaguchi, Saki Kaneko, Fumiaki Sugahara, Yasunori Murakami

**Affiliations:** 1 Graduate School of Science and Engineering, Ehime University, 2-5 Bunkyo-cho, Matsuyama, 790-8577 Japan; 2 Department of Anatomy and Neuroscience, Graduate School of Medicine and Pharmaceutical Sciences, University of Toyama, Toyama, 930-0194 Japan; 3 Division of Biology, Hyogo College of Medicine, Nishinomiya, 663-8501 Japan

**Keywords:** Catfish, Craniofacial development, Peripheral nerves, Barbels, Signaling molecules, Evolution

## Abstract

**Background:**

Catfish (Siluriformes) are characterized by unique morphologies, including enlarged jaws with movable barbels and taste buds covering the entire body surface. Evolution of these characteristics was a crucial step in their adaptive radiation to freshwater environments. However, the developmental processes of the catfish craniofacial region and taste buds remain to be elucidated; moreover, little is known about the molecular mechanisms underlying the morphogenesis of these structures.

**Results:**

In Amur catfish (*Silurus asotus*), three pairs of barbel primordia are formed by 2 days post-fertilization (dpf). Innervation of the peripheral nerves and formation of muscle precursors are also established during early development. Taste buds from the oral region to the body trunk are formed by 4 dpf. We then isolated catfish cognates *Shh* (*SaShh*) and *Fgf8* (*SaFgf8*), which are expressed in maxillary barbel primordium at 1–2 dpf. Further, SHH signal inhibition induces reduction of mandibular barbels with abnormal morphology of skeletal elements, whereas it causes no apparent abnormality in the trigeminal and facial nerve morphology. We also found that mandibular barbel lengths and number of taste buds are reduced by FGF inhibition, as seen in SHH signal inhibition. However, unlike with SHH inhibition, the abnormal morphology of the trigeminal and facial nerves was observed in FGF signal-inhibited embryos.

**Conclusion:**

The developmental processes of Amur catfish are consistent with those reported for other catfish species. Thus, developmental aspects of craniofacial structures and taste buds may be conserved in Siluriformes. Our findings also suggest that SHH signaling plays a crucial role in the formation of barbels and taste buds, without affecting nerve projection, while FGF signaling is required for the development of barbels, taste buds, and branchial nerves. Thus, SHH and FGF signaling plays key roles in the ontogenesis and evolution of some catfish-specific characteristics.

**Electronic supplementary material:**

The online version of this article (10.1186/s40851-019-0135-1) contains supplementary material, which is available to authorized users.

## Introduction

**Fig. 1:**
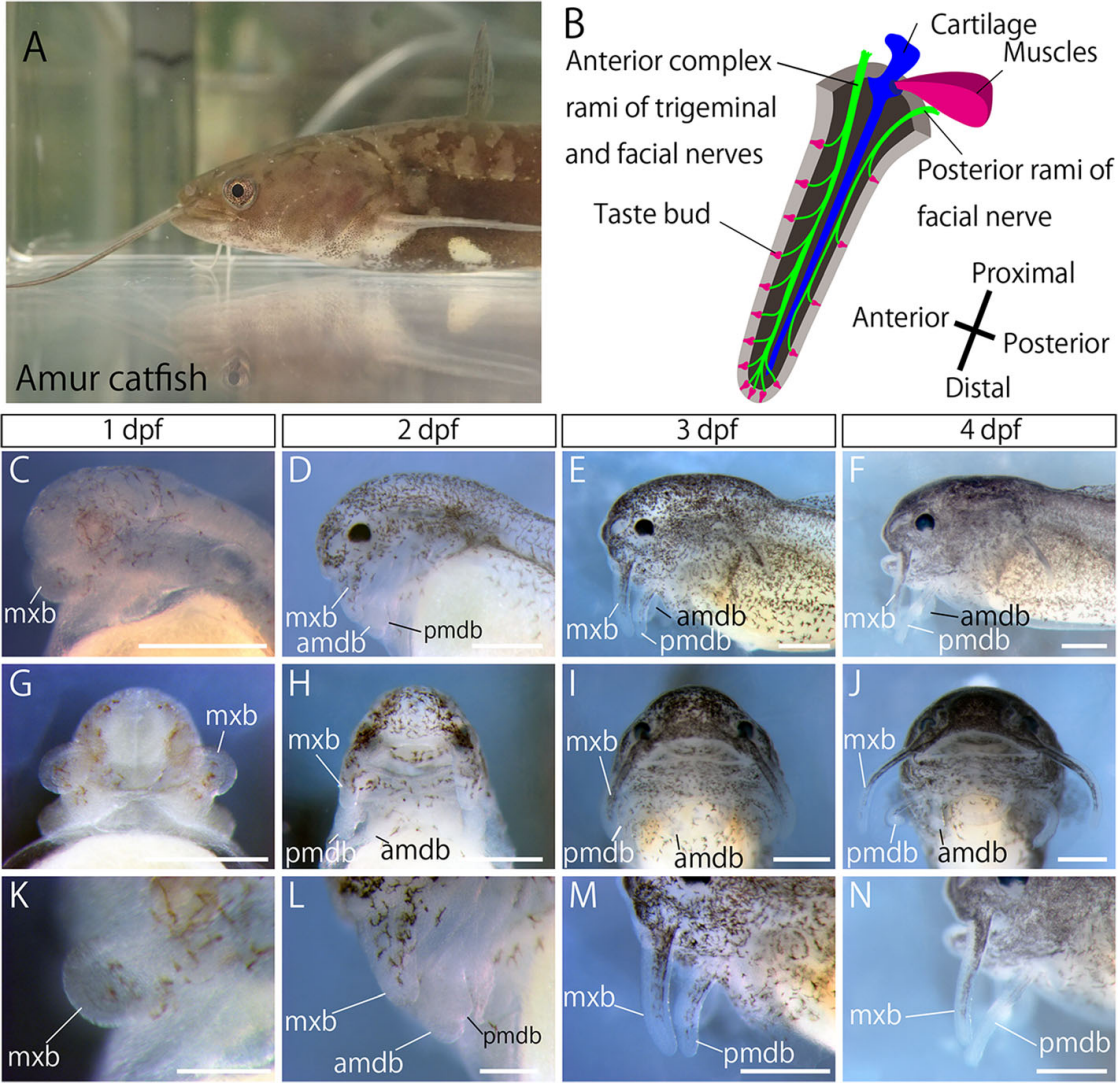
Maxillary barbel and Craniofacial development of Amur catfish. **a** Lateral view of Amur catfish. **b** Schematic drawing of the maxillary barbel. This structure is composed of taste buds, blood vessels (not shown), cartilages, muscles, and peripheral nerves. The peripheral nerves include the anterior complex rami of trigeminal and facial nerves and posterior ramus of the facial nerve. Anterior is left and the proximal side is the upper side. Catfish embryos at 1 dpf (**c**, **g**, **k**), 2 dpf (**d**, **h**, **l**), 3 dpf (**e**, **i**, **m**), and 4 dpf (**f**, **j**, **n**) observed using optical macroscopy. Lateral views are shown in **c**–**f** and **k**–**n**. Ventral views are shown in **g**–**j**. amdb, anterior mandibular barbel; mxb, maxillary barbel; pmdb, posterior mandibular barbel. Scale bars in **c**–**j**, **m**, **n** are 500 μm, and 200 μm in **i**, **j**

Extant fish are both largely diverse (approximately 32,000 species) and widely distributed across both saltwater and freshwater environments. Among these, catfish (Siluriformes) are highly diversified into over 3730 living species, which are distributed throughout every continent except Antarctica [1–4]. During adaptive radiation, catfish appear to have evolved a unique craniofacial morphology, including expanded jaws, well-developed sensory barbels, and taste buds distributed across the body surface (Fig. 1a) [5].

Barbels are generally known as tentacular sensory structures in aquatic vertebrates, including fishes, amphibians, and reptiles [6]. Fish barbels are able to detect tactile and/or chemosensory stimuli and are composed of cartilages, blood vessels, taste buds, and/or tentacle muscles attached to the barbel cartilage (Fig. 1b) [6, 7]. Generally, barbels are innervated by peripheral nerves, including the trigeminal and facial nerves. In catfish, normally up to four pairs of head barbels are observed, including one nasal, one maxillary, and two mandibular [4]. Several species of Cypriniformes, including zebrafish *Danio rerio*, also have barbels and the development and regeneration of the zebrafish maxillary barbel (ZMB) have been studied (e.g., the early ZMB bud appears at the late larval or juvenile stages, approximately 30–40 days post-fertilization [dpf]) [8]. In contrast, unlike in Cypriniformes, the barbel primordia of Siluriformes appear in the early developmental stage [6, 8–10]. Generally, the maxillary barbel primordium appears earlier than the other two pairs of mandibular barbel primordia [6, 9, 10]. Recently, comparative analysis using genome sequences between barbeled catfish and barbel-less catfish revealed that chemokine C-C motif ligand 33 (*ccl33*) plays a key role in barbel development and regeneration [11]. However, the molecular signals underlying barbel formation remain obscure. Previous studies have shown that several morphogens (signaling molecules) are involved in the formation of craniofacial elements [12–18]. Among these molecules, SHH and FGF8, whcih are members of families of paracrine signaling factors, act as key regulators of craniofacial development in vertebrates [19, 20]. Thus, it is possible that these genes play roles in the formation of catfish-specific characteristics.

In vertebrates, taste buds serve as the cranial sensory organs for the taste system. Generally, a single taste bud is an onion-shaped structure, among which there are several distinct cell types, including taste receptor (type I) cells, support (type II) cells, presynaptic (type III) cells, and basal Meckel-like cells [21–25]. Among these, cells sense bitter, sweet, umami, and salt; they are innervated by the sensory branches of seventh, ninth, and tenth cranial nerves [25–30]. During morphogenesis of mammalian taste buds, rostral papillae are derived from the oral ectoderm and caudal from the pharyngeal endoderm; molecular signals secreted from the oral endoderm may affect taste bud induction in the oral ectoderm [28, 30–33]. In the mouse rostral tongue, the SHH signal from the ectoderm regulates taste bud formation and distribution [34–37], and is also partially mediated by the Wnt/β-catenin signaling pathway [38]. In contrast, in the zebrafish pharyngeal region, the FGF signals derived from the endoderm regulates taste bud formation, with miR-200 family and Delta-Notch signaling playing important roles in this process [39]. This cumulative evidence suggests that two independent molecular mechanisms influence vertebrate taste bud development; in mammals, SHH signaling mediated by Wnt/β-catenin induces ectoderm-derived taste buds on the rostral tongue, while in fish, FGF signaling induces endoderm-derived taste buds on the pharynx. However, the developmental mechanism of catfish taste buds, which are widely distributed over the barbels and body surface, remains unclear.

## Materials and methods

### Animals

Adult Amur catfish *Silurus asotus* were collected from the Okawa and Shigenobu Rivers in Ehime, Japan, during the breeding season (May to July) (Fig. 1a). Eggs were fertilized artificially and incubated in 10% Steinberg’s solution [40] at 20–25 °C. Embryonic stages of the catfish were determined based on dpf or Armstrong’s staging of Brown bullhead *Ameiurus nebulosus* [9]. The embryos were fixed in 4% paraformaldehyde (PFA) in phosphate-buffered saline (PBS), then dehydrated in a methanol dilution series and stored in 100% methanol at − 25 °C. These studies were performed according to the guidelines for animal use of the Ehime University Animal Care Committee.

### Treatment of embryos with chemical inhibitors

First, 10 mM of cyclopamine (Calbiochem Merck, Darmstadt, Germany; CAS No. 4449-51-8) and 10 mM of SU5402 (Calbiochem Merck, Darmstadt, Germany; CAS No. 215543–92-3) were dissolved in ethanol (EtOH) and dimethyl sulfoxide (DMSO), respectively. Amur catfish embryos were treated with 5 and 10 μM of cyclopamine or SU5402 at 25 °C, after L-cysteine treatment to remove the jelly layer on the chorion of the eggs. EtOH and DMSO were used as solvent controls for cyclopamine and SU5402 treatment. For inhibitors, we placed five embryos per well in 24-well plates, which were filled with 1 ml of 10% Steinberg’s solution. Embryos were treated with inhibitors at 1 dpf and collected at 5 dpf, then fixed in 4% PFA in PBS. Since catfish embryos normally hatch at 1–2 dpf, they hatch from the eggs less than 24 h after inhibitor treatment. We did not change the solution during the treatment of inhibitors. After fixation, the length of barbels, total length, head to anal length, and jaw width were measured using a macroscope (Olympus, Tokyo, Japan).

### cDNA cloning and sequencing

Total *S. asotus* RNA was extracted from whole embryos at 1 dpf using TRIZOL reagent (Invitrogen, Carlsbad, USA). Collected RNA was used for reverse transcription (RT) to obtain cDNA, using random hexamers. Polymerase chain reaction (PCR) was next performed using embryonic cDNA as a template to amplify fragments of homologous *Fgf8* and *Shh* catfish genes. To obtain clones of catfish *Fgf8* and *Shh*, the following primers were used:

- *SaFgf8*,
- 5′-ACCTACCAGCTTTACAGCCG-3′ (forward),
- 5′-CTTCATGAAGTGGACTTCCC-3′ (reverse);
- *SaShh*,
- 5′-GCCAGCGGCAGATACGAGGGCAA-3′ (forward),
- 5′-GGTTCTGTGAACATGATGAAGTCG-3′ (reverse).

PCR product in the agarose gel was purified using Wizard SV Gel and PCR Clean-Up System (Promega, Madison, USA) and DNA fragments cloned using pGEM-T easy (pGEM-T Easy Vector Systems, Promega, Madison, USA). The isolated clone sequences were compared with the sequences of homologous genes registered on the NCBI database. Sequence analysis revealed that cloned sequences of *Fgf8* and *Shh* exhibited 91% homology between catfish and *Astyanax mexicanus Fgf8* and 99% homology between catfish and *Oryzias latipes Shh*. DDBJ/EMBL/GenBank Accession Numbers LC473500 (*SaFgf8*) and LC473499 (*SaShh*), respectively. Phylogenetic trees were made by Maximum likelihood method using MRGA6.0 software [41] (Additional file 1: Figure S1).

### Whole-mount in situ hybridization and immunohistochemistry

In situ hybridization was performed as previously described [42, 43]. For the preparation of sections, stained samples were cut into 50 μm using a vibratome after embedding in 5% agar, and were then observed under a microscope (M165FC; Leica, Wetzlar, Germany). Taste buds were marked by immunohistochemistry using anti-calretinin antibody (GeneTex, San Antonio, USA). Peripheral nerve axons were stained using anti-acetylated tubulin antibody (T-6793, diluted 1/1000; Sigma-Aldrich, St. Louis, USA). Developing muscles were visualized by anti-MF20 antibody (obtained from the Developmental Studies Hybridoma Bank, University of Iowa; diluted 1/100). As the secondary antibody, we used Alexa Fluor 488 goat anti-mouse IgG (H + L; A11001; Invitrogen) and Alexa Fluor 555 goat anti-mouse IgG (H + L; A21422; Invitrogen, Carlsbad, USA). Nuclei were labeled with DAPI (D9564, 1 mg/mL, Sigma-Aldrich, St. Louis, USA). For whole-mount immunostaining, embryos were prepared as previously described [44, 45]. The stained specimens were observed using confocal microscopy (LSM 510 META, Carl Zeiss, Oberkochen, Germany) or fluorescence macroscopy (Lumar V12; Carl Zeiss, Oberkochen, Germany).

### Cartilage staining

Fixed embryos were treated overnight with a mixture of 30% hydrogen peroxide and methanol (9,1) and then with staining solution (0.015% Alcian blue, 0.005% Alizarin red, 5% AcOH, and 70% EtOH). After staining, the samples were treated twice with 0.1% KOH containing 20% glycerol and cleared in a glycerol series. The samples were observed with macroscopy (Olympus, Tokyo, Japan).

### Scanning electron microscopy

For detailed observations of the external morphology of signal-inhibited embryos, specimens hydrated and postfixed with 1% OsO_4_ were dehydrated through a graded ethyl alcohol series and dried with a CO_2_ critical point dryer (CPD300, LEICA, Wetzlar, Germany). Dried specimens were mounted on a double-sided adhesive conductive carbon tape on copper stubs and coated with platinum in an ion sputter (Ion Sputter JFC-1600, JEOL, Tokyo, Japan) and observed under a scanning electron microscope (JSM-5600, JEOL, Tokyo, Japan).

## Results

### Overview of embryogenesis

To identify the developmental process of catfish-specific characteristics, we first observed their embryonic development. By 1 dpf, fertilized eggs continued cell cleavage and formed the embryonic body. In the tail bud stage, embryos began spontaneous swimming within their chorions. At 1 dpf, embryo organogenesis and hatching occurred. At 2–3 dpf, embryos became larvae and swam in water. At 4 dpf or later, body size increased and larvae became juveniles. This developmental process was similar to that of Brown bullheads (*Ameiurus nebulosus*) and channel catfish (*Ictalurus punctatus*), described in previous studies, although the time course of Amur catfish was faster compared with those catfish, which hatch at 5 dpf [9, 10].

### Morphogenesis of barbels

At 1 dpf, a pair of barbel primordia appeared on the lateral sides of the anterior pharyngeal region (Fig. 1c, g). These maxillary barbel primordia were covered by epidermis and appeared to be filled with mesenchymal tissue (Fig. 1k). Subsequently, two pairs of mandibular barbel primordia appeared on the mandibular region at 2 dpf (Fig. 1d, h, l). Both maxillary and mandibular barbel primordia increased in length over the course of development (Fig. 1e, f, i, j, m, n).

### Development of taste buds

**Fig. 2:**
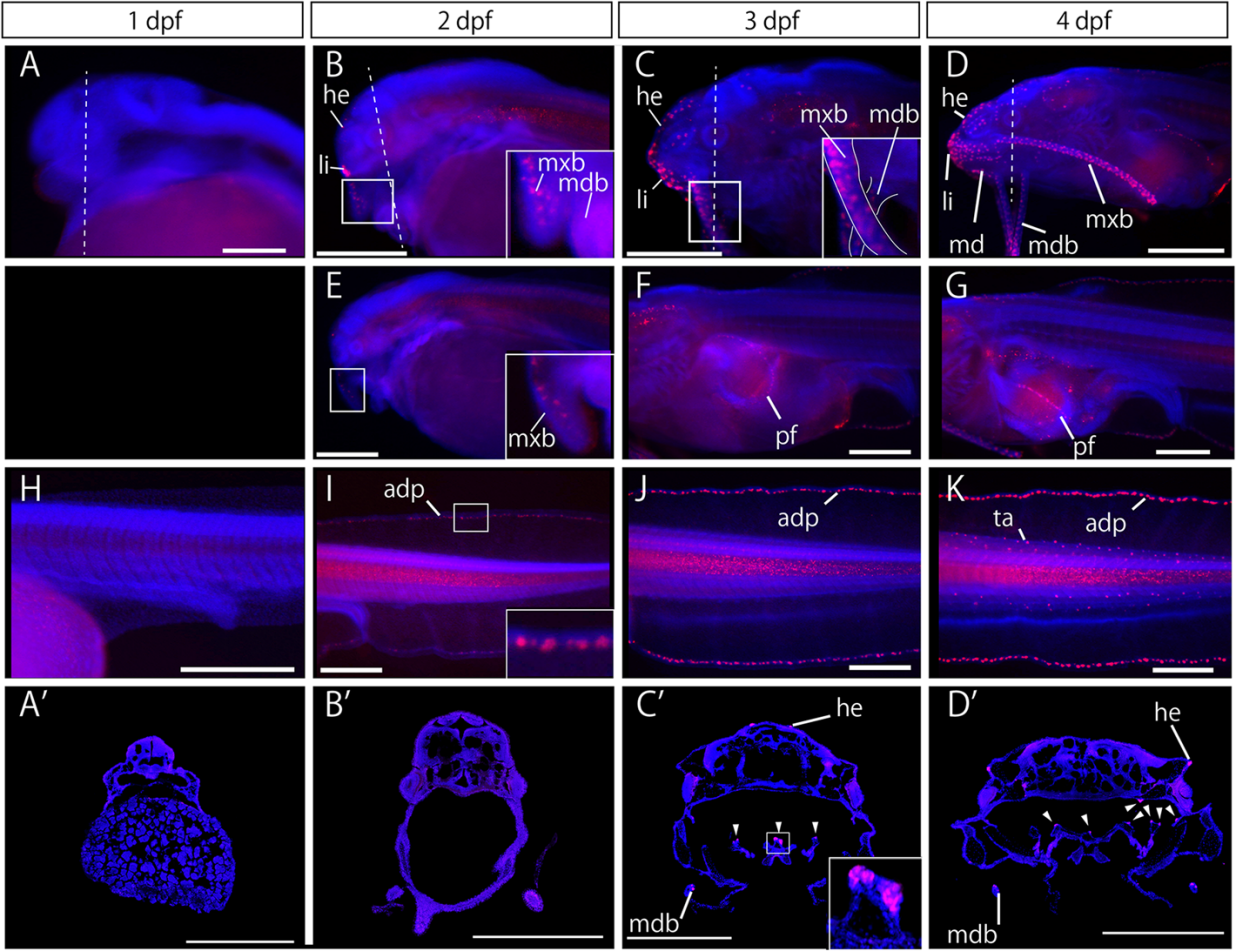
Taste bud development of Amur catfish. Catfish embryos at 1 dpf (a, h, a′), 2 dpf (b, e, i, b′), 3 dpf (c, f, j, c′), and 4 dpf (d, g, k, d′) observed using fluorescent macroscopy. Lateral views are shown in a–k. Taste buds of the head (a–g) and trunk and tail (h-k) regions were visualized by anti-calretinin antibody (in red). Coronal sections of the oral cavity are shown in a′–d′. The dashed line in a–d indicates the cutting plane in a′–d′. Taste buds in the oral cavity are indicated by arrowheads. Blue staining shows nucleus labeled by DAPI. Insets in b, c, e and c′ indicate high magnification images of boxes in b, c, e and c′. adp, adipose fin; he, head region; li, lip; mdb, mandibular barbel; mxb, maxillary barbel; pf, pectoral fin; ta, tail region. Anterior is left in a–k. Scale bars in b–g, i–k and b′-d′ are 500 μm, and 200 μm in a, h and a′

We defined the taste buds as gustatory sensory cells marked by anti-calretinin antibody. Taste buds could not be observed at 1 dpf (Fig. 2a, h), and initially appeared on the rostral side of the maxillary barbel primordium at 2 dpf (Fig. 2b, e). In addition, they were found on the upper lip and the adipose fin (Fig. 2b, i). However, taste buds were not observed in the oral cavity by 2 dpf (Fig. 2a′, b′). At 3 dpf and at later stages, taste buds appeared and increased in number on the caudal side of the maxillary barbel primordium, pectoral fin, two pairs of mandibular barbel primordia, and oral cavity (Fig. 2c, d, f, g, j, k, c′, d′).

### Development of peripheral nerves

**Fig. 3:**
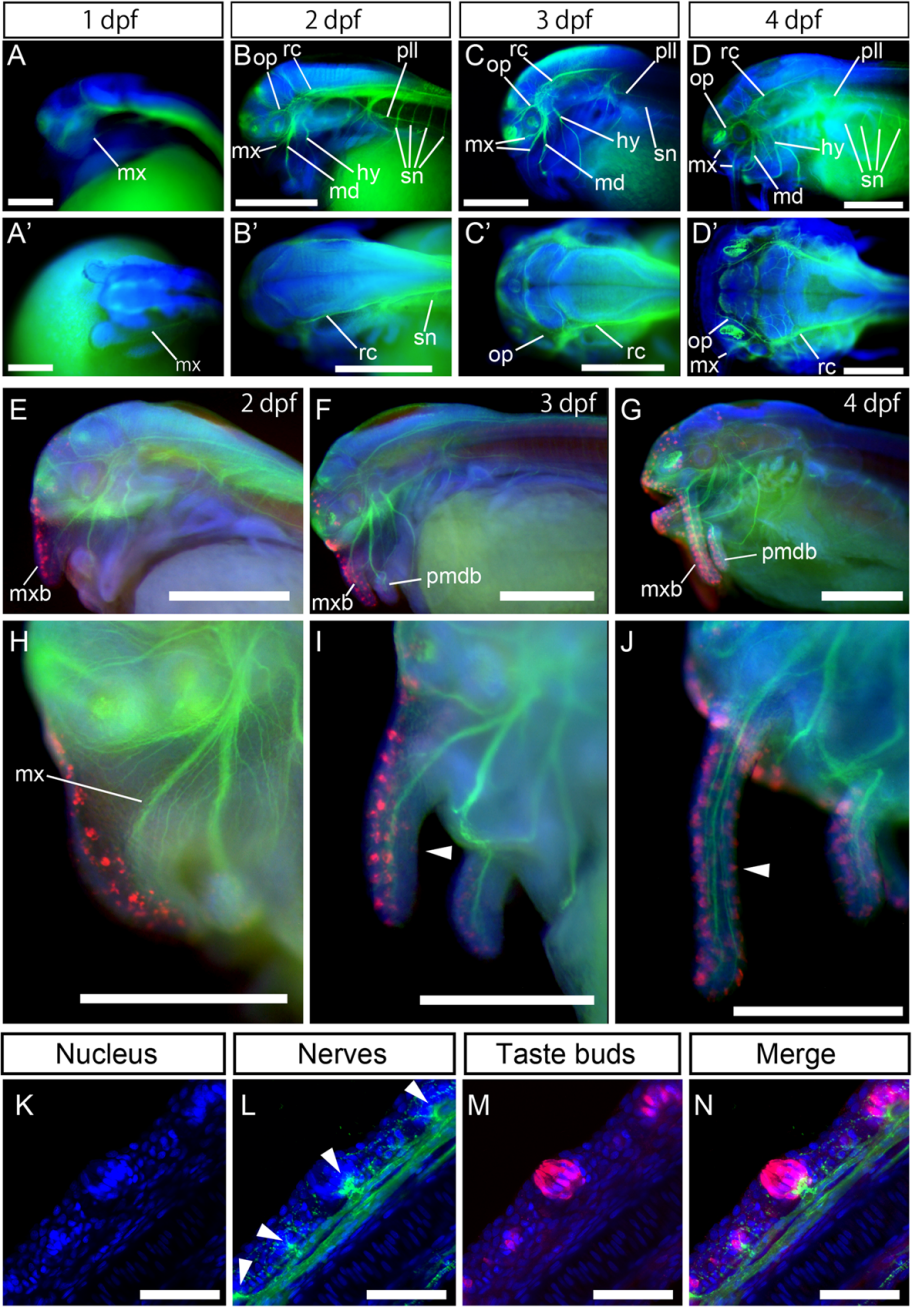
Development of peripheral nerves in Amur catfish. Peripheral nerve axons stained by anti-acetylated tubulin antibody are shown in in green. Blue staining shows nucleus labeled by DAPI. Lateral views are shown in a–d and e–j. Dorsal views are shown in a′–d′. Distribution of taste buds and innervation of peripheral nerves are shown in e–n. Arrowheads in i, j show maxillary barbels. Morphological relationships between taste buds and facial nerves on the barbel are shown in k–n. Peripheral nerve axons and taste buds were stained using anti-acetylated tubulin (in green) and anti-calretinin (in red) antibodies, respectively. Taste buds on catfish barbel are innervated by facial nerves and the nerve endings represent a specific “loop” (l, arrowheads). amdb, anterior mandibular barbel; mxb, maxillary barbel; hy, hyoid ramus of facial and anterior lateral line nerves; md, mandibular ramus of trigeminal and facial nerves; mx, maxillary ramus of trigeminal and facial nerves; op, ophthalmic ramus of trigeminal and facial nerves; pll, posterior lateral line nerve; pmdb, posterior mandibular barbel; rc, recurrent ramus of the facial nerve; sn, spinal nerves; Anterior is left in all images. Scale bars in a is 200 μm, 500 μm in b–j, and 50 μm in k–n

We did not observe any peripheral nerve axons at 1 dpf (Fig. 3a, a′). At 2 dpf, the maxillary nerve, consisting of facial and trigeminal nerve branches, had already entered the maxillary barbel primordium, on which taste buds could be observed (Fig. 3b, b′, e, h). A recurrent ramus of facial nerves extending toward the posterior side appeared at 2 dpf (Fig. 3b, b′). The posterior lateral line nerve and spinal nerves were also observed at this stage (Fig. 3b, b′). At 3–4 dpf, development of peripheral nerves continued (Fig. 3c, c′, d, d′, f, g, i, j). Among these, the maxillary nerve reached the tip of the developing maxillary barbel (Fig. 3i, j). At 5 dpf, facial nerve axons in the maxillary barbel made “loop-shaped” endings in the basal region of the taste buds (Fig. 3k-n).

### Development of cartilages and muscles

**Fig. 4:**
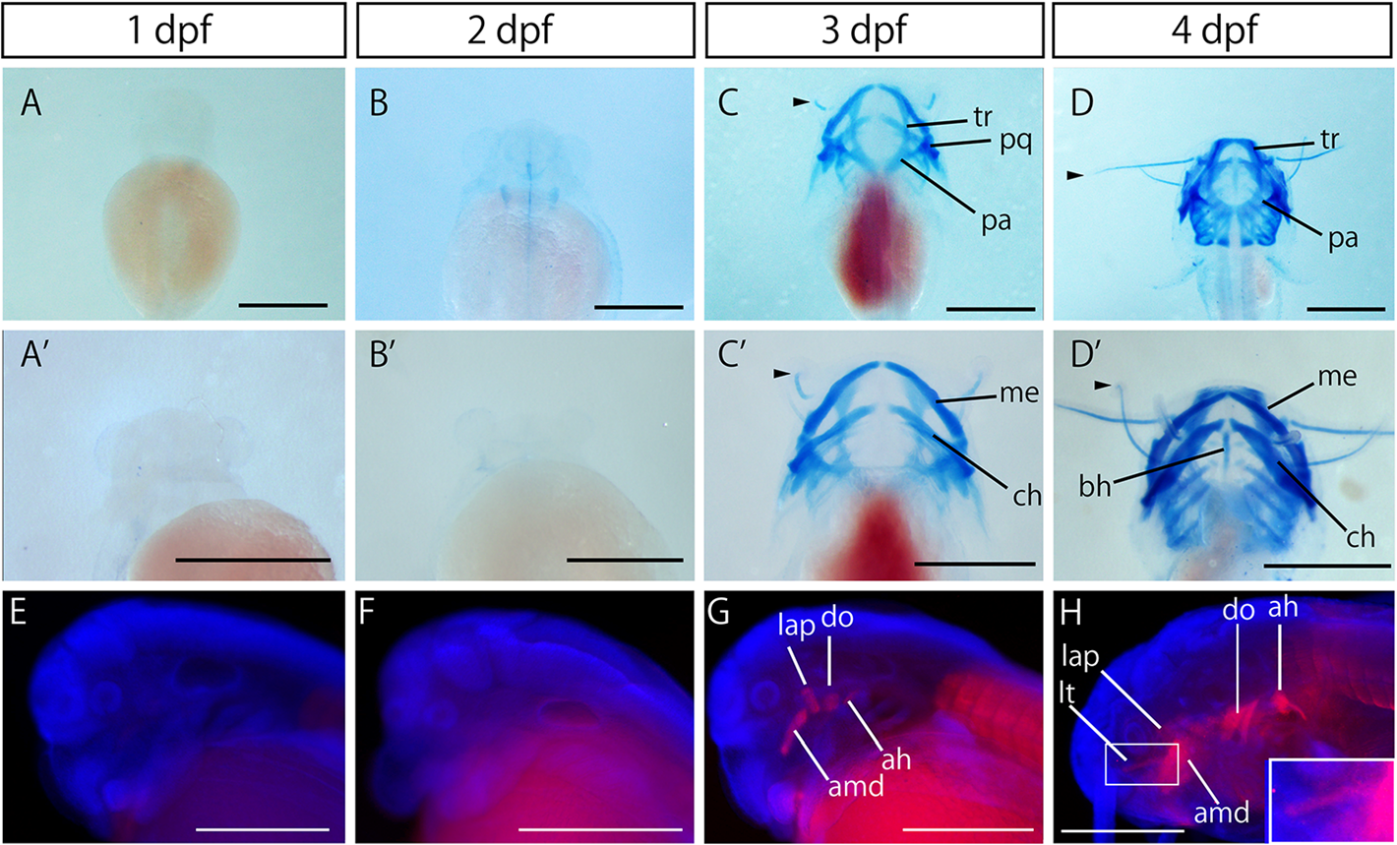
Development of cartilages and muscles in Amur catfish. Cartilages stained by Alcian blue are shown in a–d. Myosin filaments stained by immunohistochemistry using anti-MF20 antibody are shown in e–h (in red). Blue staining in e–h shows nucleus labeled by DAPI. Dorsal views are shown in a–d. Ventral views are shown in a′–d′. Lateral views are shown in e–h. Arrowheads in c, c′ and d′ indicate barbal cartilages. The inset in h indicates high magnification images of the box in h. ah, adductor hyomandibulae; amd, adductor mandibulae; bh, basihyal cartilage; ch, ceratohyal cartilage; do, dilator operculi; lap, levator arcus palatini; lt, levator tentacle; me, Meckel’s cartilage; pa, parachordal cartilage; pq, palatoquadrate cartilage; tr, trabecular cartilage. Anterior is the upper side in a–d and left in e-h. Scale bars are 1 mm in a–d, and 500 μm in e–h

We could not identify any cartilages by 2 dpf (Fig. 4a, a′, b, b′). At 3 dpf, Meckel’s, parachordal, ceratohyal, palatoquadrate, basihyal and trabecular cartilages appeared (Fig. 4c, c′). These cranial cartilages continued to grow at 4 dpf and into later stages (Fig. 4d, d′). The maxillary and mandibular barbel cartilages reached the distal end by 3 dpf (arrowheads in Fig. 4c, c′, d, d′).

Concerning muscles, no myosin filaments could be identified at either 1 or 2 dpf (Fig. 4e, f). Cranial muscles, including adductor hyomandibulae, adductor mandibulae, dilator opercula and levator arcus palatini, appeared at 3 dpf (Fig. 4g). The levator tentacle (lt) was observed at the base of the maxillary barbel at 4 dpf (Fig. 4h).

### Expression patterns of signaling molecules

**Fig. 5:**
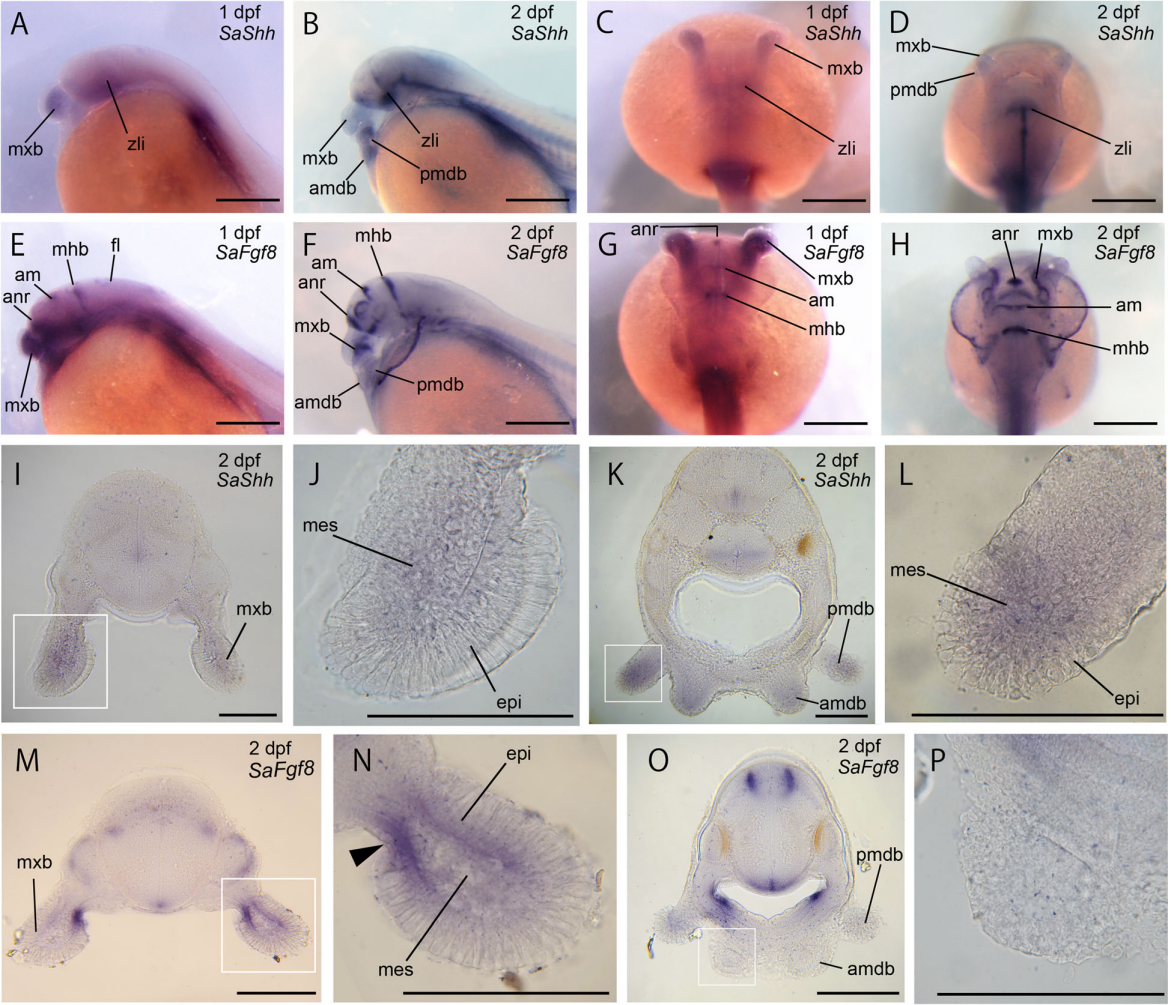
Expression pattern of catfish orthologs of *Shh* (*SaShh*) and *Fgf8* (*SaFgf8*). **a**–**d**, transcripts of *SaShh* in Amur catfish embryos at 1 dpf (**a**, **c**), 2 dpf (**b**, **d**) are shown. **e**–**h**, transcripts of *SaFgf8* in Amur catfish embryos at 1 dpf (**e**, **g**), 2 dpf (**f**, **h**) are shown. **a**, **b**, **e**, **f** are lateral views. **c**, **d**, **g**, **h** are dorsal views. Anterior is left in **a**, **b**, **e**, **f** and the upper side in **c**, **d**, **g**, **h**. **i**–**p**, coronal sections of Amur catfish embryos at 2 dpf. **i**, **m** and **k**, **o** are the slice of anterior and posterior head, respectively. **i**-**l**, transcripts of *SaShh* are shown. **m**–**p**, transcripts of *SaFgf8* are shown. **j**, **l**, **n** and **p** are high magnification images of boxes in **i**, **k**, **m** and **o**. Arrowhead indicates *SaFgf8* expression domain at the medial epidermal layer. am, anterior midbrain; amdb, anterior mandibular barbel; anr, anterior neural ridge; epi, epidermis; fl, facial lobe; mes, mesenchyme; mhb, midbrain–hindbrain boundary; mxb, maxillary barbel; pmdb, posterior mandibular barbel; zli, zona limitans intrathalamica. Scale bars are 500 μm in A–G, and 200 μm in **i**–**p**

To identify the role of signaling molecules in catfish morphogenesis, we initially studied the expression pattern of the presumptive catfish ortholog of *Shh* (*SaShh*) (Fig. 5a-d). At 1 dpf, *SaShh* mRNA was expressed in the mesenchyme of maxillary barbel primordia, the ventral side of the neural tube, and the presumptive zona limitans intrathalamica (zli) (Fig. 5a, c). At 2 dpf, the *SaShh* expression domain was sustained in zli (Fig. 5b), while additional expression domains appeared in two pairs of mandibular barbel primordia, where *SaShh* was expressed in the mesenchyme (Fig. 5d, i-l). Later *SaShh* expression in both maxillary and mandibular barbels was not detected (data not shown).

We then studied the expression pattern of the presumptive catfish ortholog of *Fgf8* (*SaFgf8*) (Fig. 5e-h). At 1 dpf, *SaFgf8* transcripts were observed in many parts of the neural tube: the anterior neural ridge (anr), anterior midbrain and midbrain–hindbrain boundary (mhb) (Fig. 5e, g). *SaFgf8* transcripts were also detected in the maxillary barbel primordium (Fig. 5e, g). At 2 dpf, *SaFgf8* mRNA was still expressed in anr, anterior midbrain, and mhb (Fig. 5f, h). At this stage, the *SaFgf8* expression domain was restricted to the proximal maxillary barbel, where it was located at the medial region of the epidermal layer (Fig. 5f, h, m, arrowhead in n). Interestingly, unlike the maxillary barbel, expression of *SaFgf8* could not be detected in the anterior mandibular barbel primordium at 2 dpf (Fig. 5f, o, p). At 3 dpf and later stages, *SaFgf8* expression was not observed in either maxillary or mandibular barbels (data not shown).

### Inhibition of SHH and FGF signaling: defects in craniofacial morphology

**Fig. 6:**
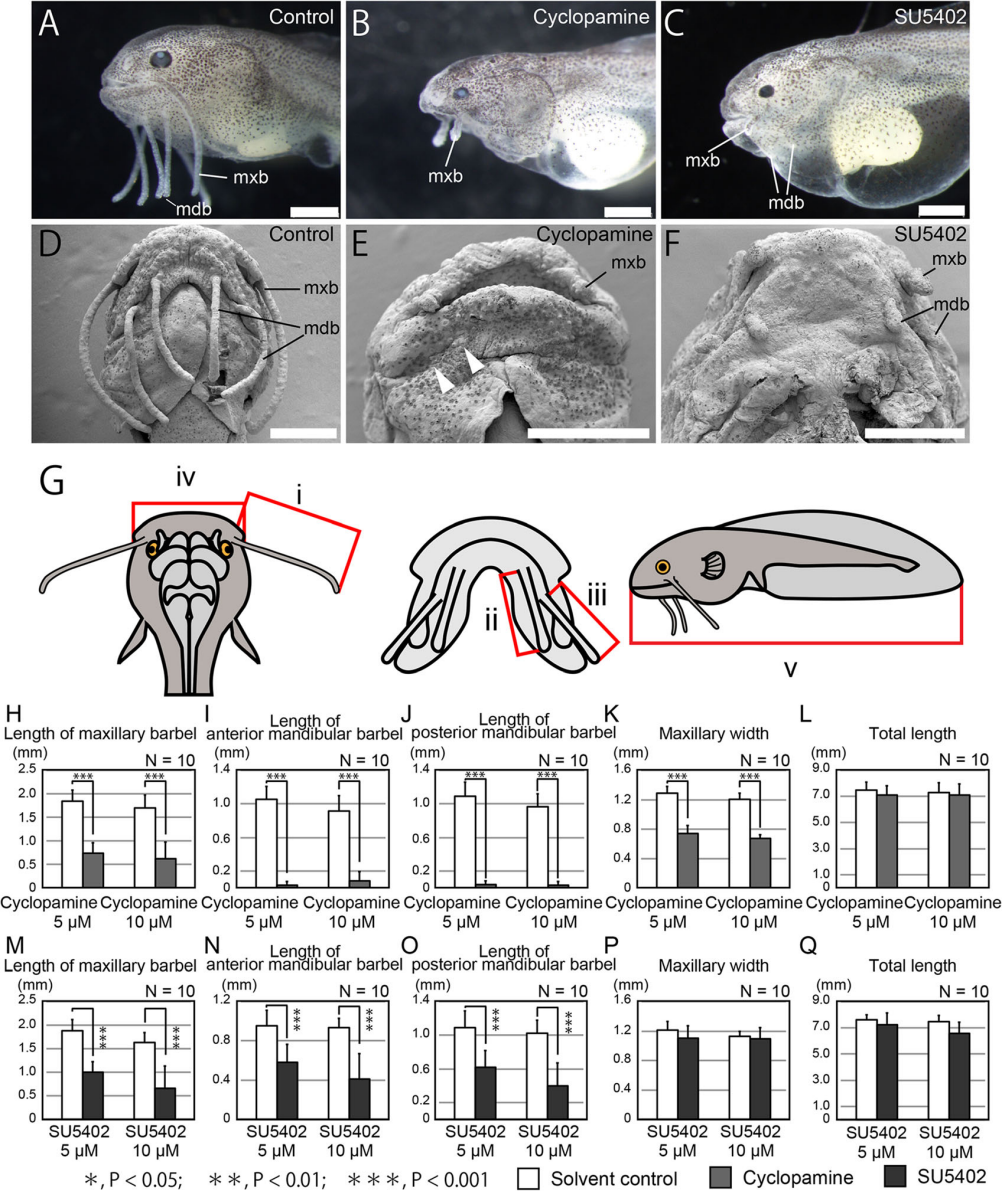
Effects of SHH and FGF signal inhibitors on morphology of catfish embryos**.** Specimens were observed by microscopy (**a**–**c**) and SEM (**d**–**f**). In the developing catfish, SHH and FGF inhibitors (10 μM of Cyclopamine in **b**, **e** and 10 μM of SU5402 in **c**, **f**) affect morphogenesis of the craniofacial region. SHH inhibitor induced malformation of mandibular apparatus and reduced the length of maxillary barbels (**b**, **e**). In addition, mandibular barbels disappeared in cyclopamine-treated embryos (arrowheads in **e**). FGF inhibitor caused malformation of maxillary and mandibular barbels (**c**, **f**), whereas the craniofacial morphology appeared normal. **g** shows schematics of the dorsal (left), ventral (middle), and lateral (right) sides of the catfish. i–v are morphological cues used for quantification: i, length of the maxillary barbel; ii, length of the anterior mandibular barbel; iii, length of the posterior mandibular barbel; iv, width of the maxillary region; v, total length. **h**–**q**, quantification of the length of barbels (**h**–**j**, **m**–**o**), width of maxillary region (**k**, **p**), and total length (**l**, **q**) in cyclopamine-treated embryos (**h**–**l**) and SU5402-treated embryos (**m**–**q**) at 5 dpf. Inhibition of SHH induced significant abnormality in both maxillary and mandibular barbels (**h**–**j**) and maxillary apparatus (**k**). However, cyclopamine did not affect total length (**l**). Inhibition of FGFs significantly induced abnormality in both maxillary and mandibular barbels (**m**–**o**), while maxillary width and total length appeared normal (**p**, **q**). Error bars are standard deviations (SD). Data denoted by the same letter do not differ significantly (* *P* > 0.05; ** *P* > 0.01; *** *P* > 0.001) based on Tukey’s HSD test or the Steel–Dwass test after analysis of homogeneity of variance with Bartlett’s test. mdb, mandibular barbel; mxb, maxillary barbel. Anterior is left in **a**–**c** and the upper side in **d**–**f**. Scale bars are 500 μm

To identify the molecular mechanisms underlying formation of the catfish craniofacial region, we administered cyclopamine, a SHH signal inhibitor [46, 47], to catfish embryos from 1 dpf to 5 dpf. We found severe defects in the craniofacial region compared with those of the solvent control, with the length of the maxillary barbel shortening in cyclopamine-treated embryos (Fig. 6a, b, d, e). Tukey’s HSD test indicated that the length of the maxillary barbel was significantly shorter than that of the control group (Fig. 6h). Furthermore, two pairs of mandibular barbels were almost absent in cyclopamine-treated embryos (Fig. 6e, i, j). The maxillary width was significantly smaller than that of the control embryos (Fig. 6k). However, total body length appeared unaffected (Fig. 6l). These results suggest that SHH signaling is required for the development of the craniofacial region, including both maxillary and mandibular barbels.

As *SaFgf8* was strongly expressed in the developing maxillary barbel, we speculated that the FGF signal plays a crucial role in the formation of barbels. Thus, we applied SU5402, a potent FGF receptor inhibitor [48], to catfish embryos from 1 dpf to 5 dpf. Although the maxillary width and total body length were unaffected by SU5402 treatment (Fig. 6 p, q), the lengths of three pairs of barbels were significantly shorter compared with those of the solvent control (Fig. 6 a, c, d, f, m-o).

### Peripheral nerve morphology in SHH and FGF signal inhibitor-treated embryos

**Fig. 7:**
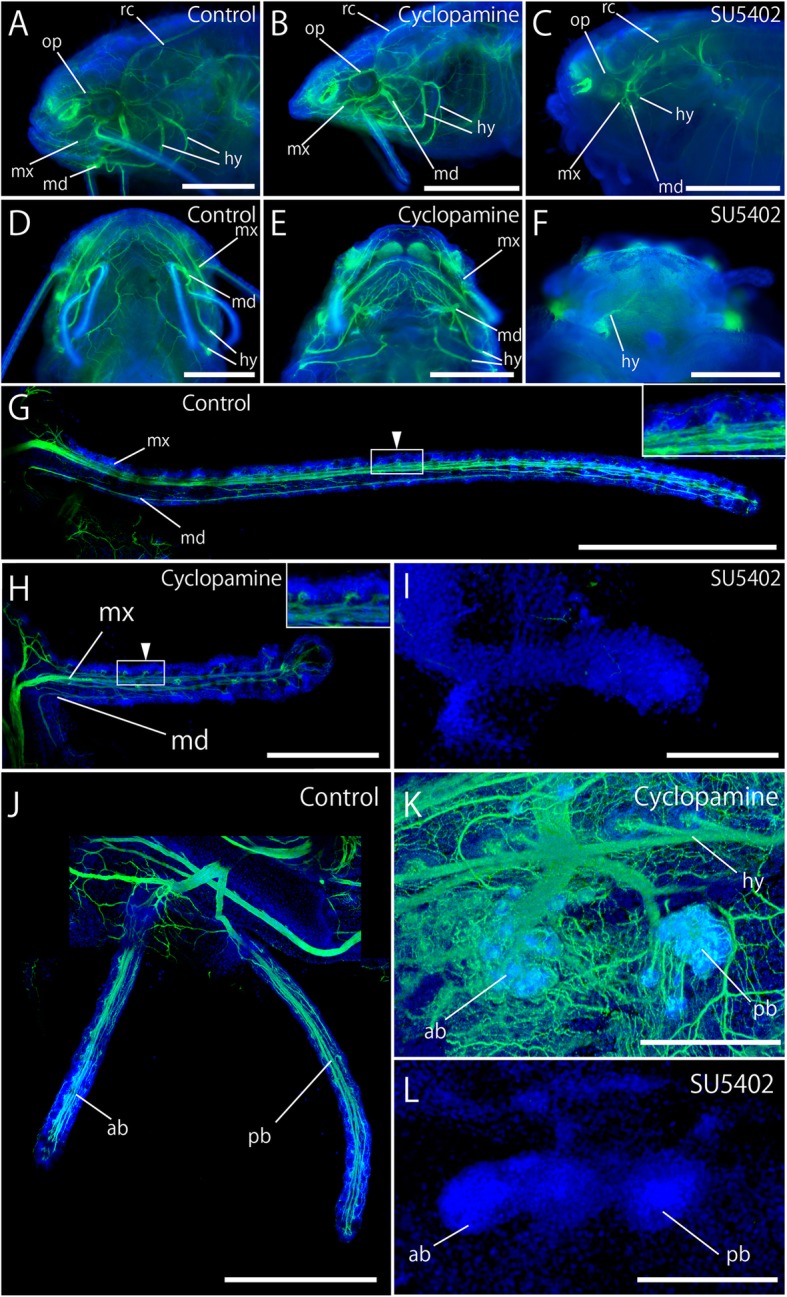
Effects of SHH and FGF inhibitors on peripheral nerve morphology**.** Peripheral nerves (in green) were visualized by anti-acetylated tubulin antibody. Blue staining shows nucleus labeled by DAPI. **a**–**c** are lateral and (**d**–**f**) are ventral. Cyclopamine-treated embryos (**b**, **e**) represented apparently normal distribution of peripheral nerves compared with the control group (**a**, **d**), although their mandibular regions became smaller and mandibular barbels were absent. However, with SU5402 treatment, peripheral nerve axons were poorly developed (**c**, **f**). **g**–**l** effects of SHH and FGF inhibitors on the peripheral nerves in the maxillary (**g**–**i**) and mandibular (**j**–**l**) barbels of Amur catfish. Insets in g, h indicate high magnification images of boxes in **g**, **h**. In the control group at 5 dpf (**g**), peripheral nerves (mx and md) extended normally and reached the distal end of the barbel. In the cyclopamine-treated embryos, both mx and md entered into the maxillary and mandibular barbels and reached the distal end, consistent with the control group, although the length of the barbels shortened (**h**, **k**). However, with SU5402 treatment, the peripheral nerves failed to enter the barbels (**i**, **l**). ab, anterior mandibular barbel ramus of trigeminal and facial nerves; hy, hyoid ramus of facial and anterior lateral line nerves; md, mandibular ramus of trigeminal and facial nerves; mx, maxillary ramus of trigeminal and facial nerves; op, ophthalmic ramus of trigeminal and facial nerves; pb, posterior mandibular barbel ramus of trigeminal and facial nerves; rc, recurrent ramus of the facial nerve. Anterior is left in **a**–**c** and the upper side in **d**–**f**. In **g**, left and upper sides show proximal and anterior sides, respectively. In h and i, left and upper sides show distal and anterior sides, respectively. In **j**–**l**, upper and left sides show proximal and anterior sides, respectively. Scale bars in **a**–**g** and **j** are 500 μm, 400 μm in H, and 200 μm in **i**, **k**, and **l**

Despite the abnormal craniofacial morphology with cyclopamine treatment, rami of cranial nerves, including a recurrent ramus of the facial nerve, were normally elongated to the peripheral region compared with controls (Fig. 7a, b). In the maxillary barbel, the maxillary (mx) and mandibular (md) rami, which include the trigeminal and facial nerves, entered the maxillary barbel and innervated taste buds by making characteristic “loop-shaped” nerve endings, consistent with the control group (Fig. 7a, b, d, e, g, h, arrowheads in g, h). In the mandibular barbels, two pairs of mandibular barbel rami, which are composed of trigeminal and facial nerves, correctly reached the basal region of the barbels, consistent with the control, although the mandibular barbels showed severe reduction in the cyclopamine-treated embryos (Fig. 7j, k).

In contrast to the cyclopamine-treated embryos, peripheral nerves developed poorly in SU5402-treated embryos (Fig. 7c, f), although the recurrent ramus of the facial nerve elongated toward the trunk region, consistent with the control group (Fig. 7a, c). In the maxillary and mandibular regions, peripheral nerve axons failed to reach the basal region of the barbels (Fig. 7i, l).

### Development of taste buds in SHH and FGF signal inhibitor-treated embryos

**Fig. 8:**
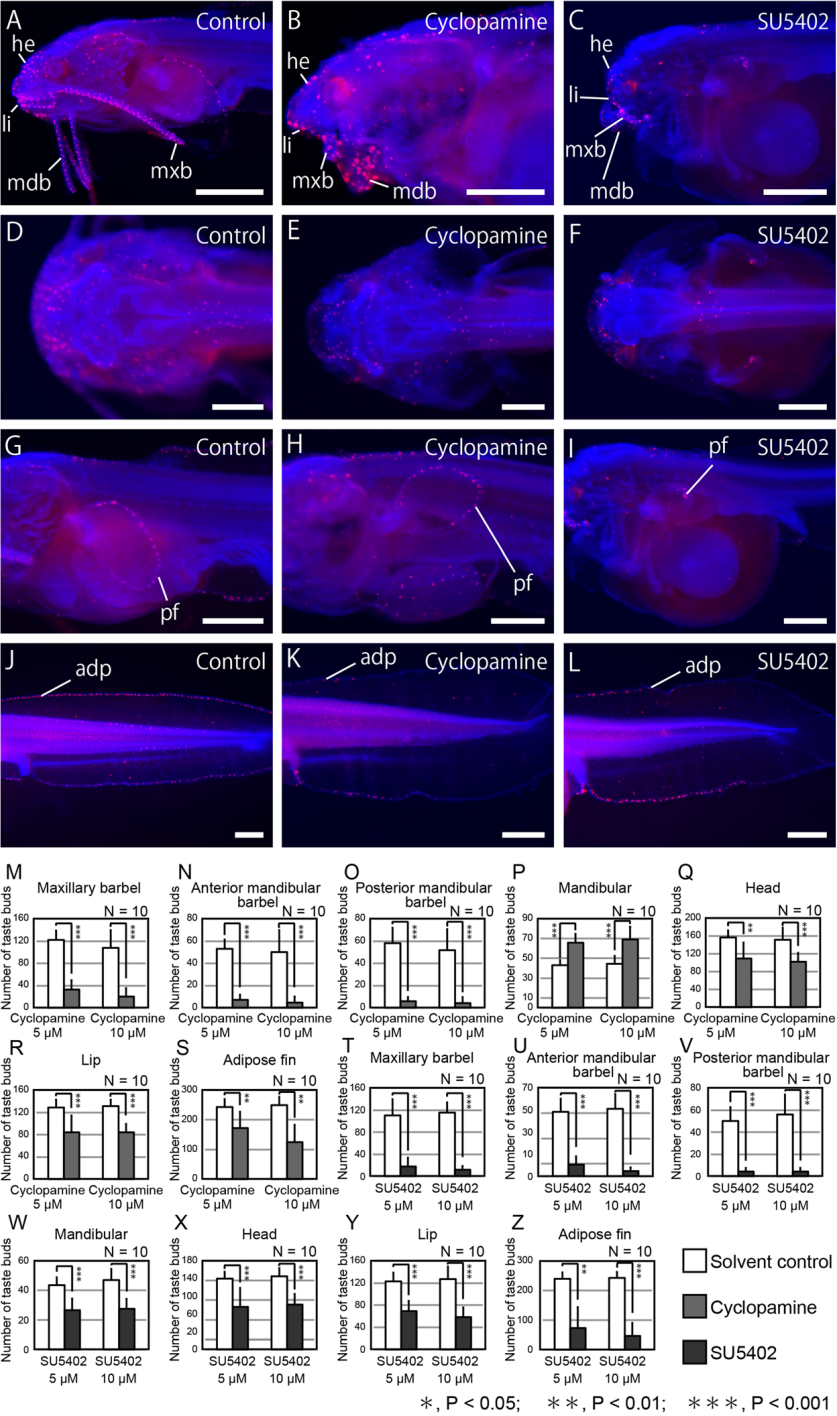
Effects of SHH and FGF inhibitors on taste bud numbers and distribution. Taste buds on the body surface, including the craniofacial region (in green) are visualized by anti-calretinin antibody. Blue staining shows nucleus labeled by DAPI. **a**–**f**, lateral (**a**–**c**) and dorsal (**d**–**f**) views of the head. **g**–**i**, lateral views of the trunk. **j**–**l**, lateral views of the tail. **a**, **d**, **g**, and **j** show control embryos at 5 dpf. **b**, **e**, **h**, and **k** show cyclopamine-treated embryos. **c**, **f**, **i**, and **l** show SU5402-treated embryos. **m**–**z**, quantification of the number of taste buds on barbels (**m**–**o**), craniofacial regions (**p**, **r**) and adipose fin (s) in cyclopamine-treated embryos. Inhibition of SHH signal significantly reduced numbers of body surface taste buds, including the craniofacial region, except for the mandibular region (**m**–**o**, **q**–**s**). In contrast, the numbers of taste buds were significantly increased in the mandibular region (**p**). **t**–**z**, quantification of the number of taste buds on barbels (**t**–**v**), craniofacial and trunk regions, including the mandibular apparatus (**w**), head (**x**), upper and lower lips (**y**), and adipose fin (**z**) in SU5402-treated embryos. Inhibition of FGF signal significantly reduced the number of taste buds on the whole of the body surface, including the craniofacial region. Error bars are standard deviation (SD). Data denoted by the same letter do not differ significantly (*P > 0.05; **P > 0.01) based on Tukey’s HSD test or the Steel–Dwass test after analysis of homogeneity of variance by Bartlett’s test. adp, adipose fin; he, head region; li, lip; mdb, mandibular barbel; mxb, maxillary barbel; pf, pectoral fin; tail region. Anterior is left in all images. Scale bars are 500 μm

We then studied the spatial distribution of taste buds on the head, trunk, adipose fin, pectoral fin, lip, mandibular, and three pairs of barbels at 5 dpf (Fig. 8). We found that taste bud distribution appeared normal in cyclopamine-treated embryos (Fig. 8a, b, d, e, g, h, j, k). However, taste buds were significantly decreased in several parts of the body (Fig. 8m–o, q–s), except in the mandibular region, where they appeared to be increased (Fig. 8b, p). These results suggest that SHH signaling regulates the number of taste buds without grossly affecting their spatial distribution.

Taste buds in SU5402-treated embryos were distributed over the entire body surface (Fig. 8c, f, i, l). However, they were significantly reduced in number on all areas observed (Fig. t–z). Taste buds were also distributed over the surface of the developing maxillary barbel (Fig. 8c, t), whereas peripheral nerve axons failed to enter into the barbel in SU5402-treated embryos (Fig. 7i). Thus, these taste buds may not be innervated by the maxillary nerve.

### Morphology of cartilages in SHH and FGF signal inhibitor-treated embryos

**Fig. 9:**
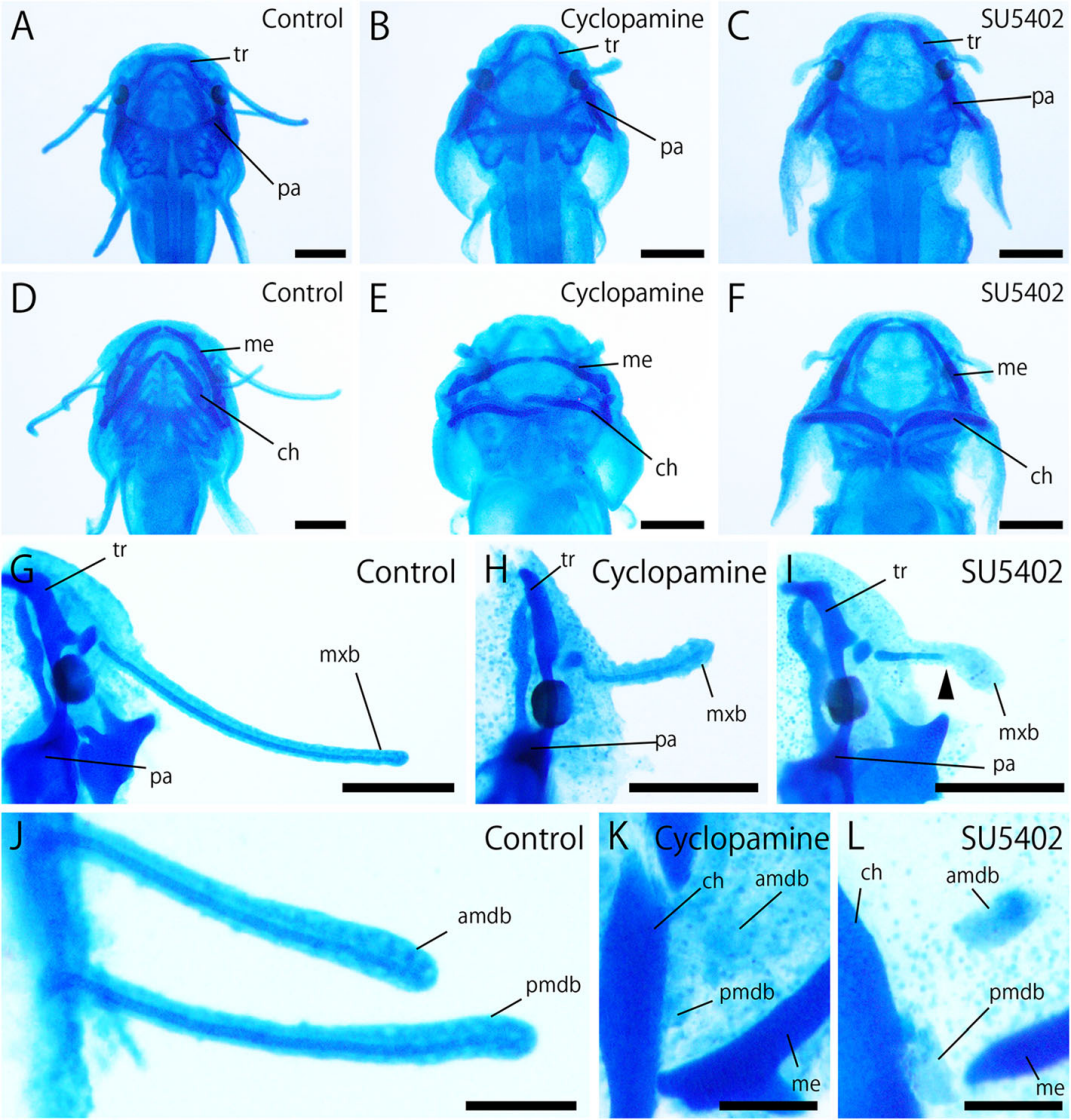
Effects of SHH and FGF signal inhibitors on cartilage formation. **a**–**f**, dorsal (**a**–**c**) and ventral (**d**–**f**) views of control (**a**, **d**), cyclopamine-treated (**b**, **e**), and SU5402-treated (**c**, **f**) embryos at 5 dpf. Inhibition of SHH caused malformation of trabecular (tr), parachordal (pa), Meckel’s (me), and ceratohyal (ch) cartilages (**b**, **e**). Inhibition of FGFs causes malformation of ceratohyal cartilage (ch), while other craniofacial cartilages appear unaffected (**c**, **f**). **g**–**l**, dorsal (**g**–**i**) and ventral (**j**–**l**) views of control (**g**, **j**), cyclopamine-treated (**h**, **k**), and SU5402-treated (**i**, **l**) embryos at 5 dpf. Cartilages in maxillary (**g**–**i**) and mandibular (**j**–**l**) barbels were visualized by Alcian blue. The cartilages of cyclopamine-treated embryos entered into the maxillary barbel and extended to the tip of the barbel, consistent with the control group (**h**), although they became thinner compared with the controls (**g**). In the mandibular region, the barbel cartilage was absent in cyclopamine-treaed group (**k**). FGF inhibition caused reduction in cartilage length in the maxillary barbel (arrowhead in **i**). In the mandibular region, barbel cartilages were severely reduced (**l**). amdb, anterior mandibular barbel; ch, ceratohyal cartilage; me, Meckel’s cartilage; mxb, maxillary barbel; pa, parachordal cartilage; pmdb, posterior mandibular barbel; tr, trabecular cartilage. Anterior is the upper side in all images and the proximal side is left in **g**–**l**. Scale bars in **a**–**i** are 500 μm and 200 μm in **j**–**l**

In cyclopamine-treated embryos, Meckel’s, Reichert’s, trabecular, and parachordal cartilages could be identified, although they had abnormal morphology compared with that of the control group (Fig. 9a-f). Cartilage in the barbels elongated until the distal end in control embryos at 5 dpf (Fig. 9g, j). In cyclopamine-treated embryos, barbel cartilage elongated to the distal end, consistent with the control group, although the whole length of the barbel was reduced (Fig. 9h). Cartilages in mandibular barbels were appeared to be absent in cyclopamine-treated embryos ((Fig. 9k). These results suggest that SHH signaling affects cartilage formation in both the pharyngeal region and barbels.

Concerning the cartilaginous tissue, developmental positions of the trabecular, parachordal, and Meckel’s cartilages appeared unaffected in SU5402-treated embryos, while ceratohyal cartilage showed abnormal position and morphology (Fig. 9f). Cartilage of the maxillary barbel failed to elongate to the distal end of the barbel in SU5402-treated embryos (Fig. 9i). Alcian blue-positive tissue could be observed in rudimentary mandibular barbels (Fig. 9l). These results indicate that FGF signaling plays an important role in the development of barbel cartilages, while it partially affects the pharyngeal cartilages.

## Discussion

### Development of catfish barbels and taste buds

We found that the catfish maxillary barbel initially appears as a bud-like process prior to the mandibular buds, then elongates anteriorly. Although the size of catfish barbel proimordia is larger than that of zebrafish maxillary barbel bud [8], the developmental time course of barbels in Amur catfish appears comparable with the ZMB. Thus, catfish and zebrafish may possess similar basic plans for the onset of barbel development. Recently, Zhou and colleagues reported that chemokine C-C motif ligand 33 (*ccl33*) acts as a key regulator for the development and regeneration of barbels in both zebrafish and catfish [11]. Therefore, this molecule may play a fundamental role in the initiation of barbel development in teleosts. In contrast, the catfish maxillary barbel bud was larger compared with that of the zebrafish and, unlike in the ZMB, appeared at the early embryonic stage. Subsequently, two pairs of mandibular barbels, which are absent in the zebrafish, appeared in bud-like processes. These results indicate that the temporal and spatial patterning of catfish barbels differs from that of zebrafish, suggesting that catfish evolved their own developmental plan for the patterning of barbels.

### Development of taste buds

We found that the developmental time course of taste buds in Amur catfish appears comparable with that of channel catfish *I. punctatus* [10], suggesting that developmental processes for taste buds are conserved in Siluriformes. In contrast, the onset of taste bud development in the catfish was earlier compared with zebrafish, which appears in the larval stage [23, 39]. We also found that taste buds in Amur catfish initially appeared on the maxillary barbel prior to other body regions, including the craniofacial epithelium, oral cavity, adipose fin, and tail region. This developmental sequence differed from that of the zebrafish, in which taste buds on the barbels formed later in development [23, 39]. Taken together, these data suggest that the developmental process of taste buds in catfish was established in Siluriformes after the split from the common ancestor of Siluriformes and Cypriniformes.

### Formation of catfish neuronal, muscular, and cartilaginous characteristics

In the present study, we found that bundles of facial and trigeminal nerves had already entered the maxillary barbel during an early developmental stage. The development and morphology of these nerves were similar to those of *I. punctatus* [10].

In Amur catfish, barbel cartilages appeared at 3 dpf prior to the formation of the levator tentacle, which attached to the maxillary barbel cartilage. This developmental time course of muscular and cartilaginous elements in the chondrocranium of Amur catfish is similar to that of the suckermouth armored catfish *Ancistrus* cf. *triradiatus* [49]. Thus, the developmental plan underlying neuron innervation and musculoskeletal patterning in the craniofacial region may be highly conserved in Siluriformes.

### Expression of *SaShh* and *SaFgf8* in catfish development

It is well known that SHH and FGF8 play fundamental roles in the development of the vertebrate craniofacial region [49–51]. Our sequence analysis revealed that cloned sequences of *Shh* exhibited 99% homology between catfish and *Oryzias latipes Shh* and that *Fgf8* exhibited 91% homology between catfish and *Astyanax mexicanus*. Moreover, expression patterns of these transcripts were highly similar to those of other vertebrates [52–56]. These results support the notion that we obtained authentic orthologs of *Shh* and *Fgf8*. *SaShh* transcripts were expressed in maxillary and mandibular barbel buds in developing catfish embryos and were downregulated in the barbel primordium at later stages. These results lead to the hypothesis that *SaShh* is involved in barbel morphogenesis during the early stages of catfish embryos. *SaFgf8,* a presumptive ortholog of *Fgf8*, was also expressed in the whole part of the developmental maxillary barbel bud at 1 dpf. At the later stage (2 dpf), expression of *SaFgf8* restricted to the epidermis in the proximal and medial sides of the maxillary barbel bud. These developmental changes of expression pattern appeared synchronized with the elongation period of the barbels (see below).

### Role of SHH in barbel development

**Fig. 10:**
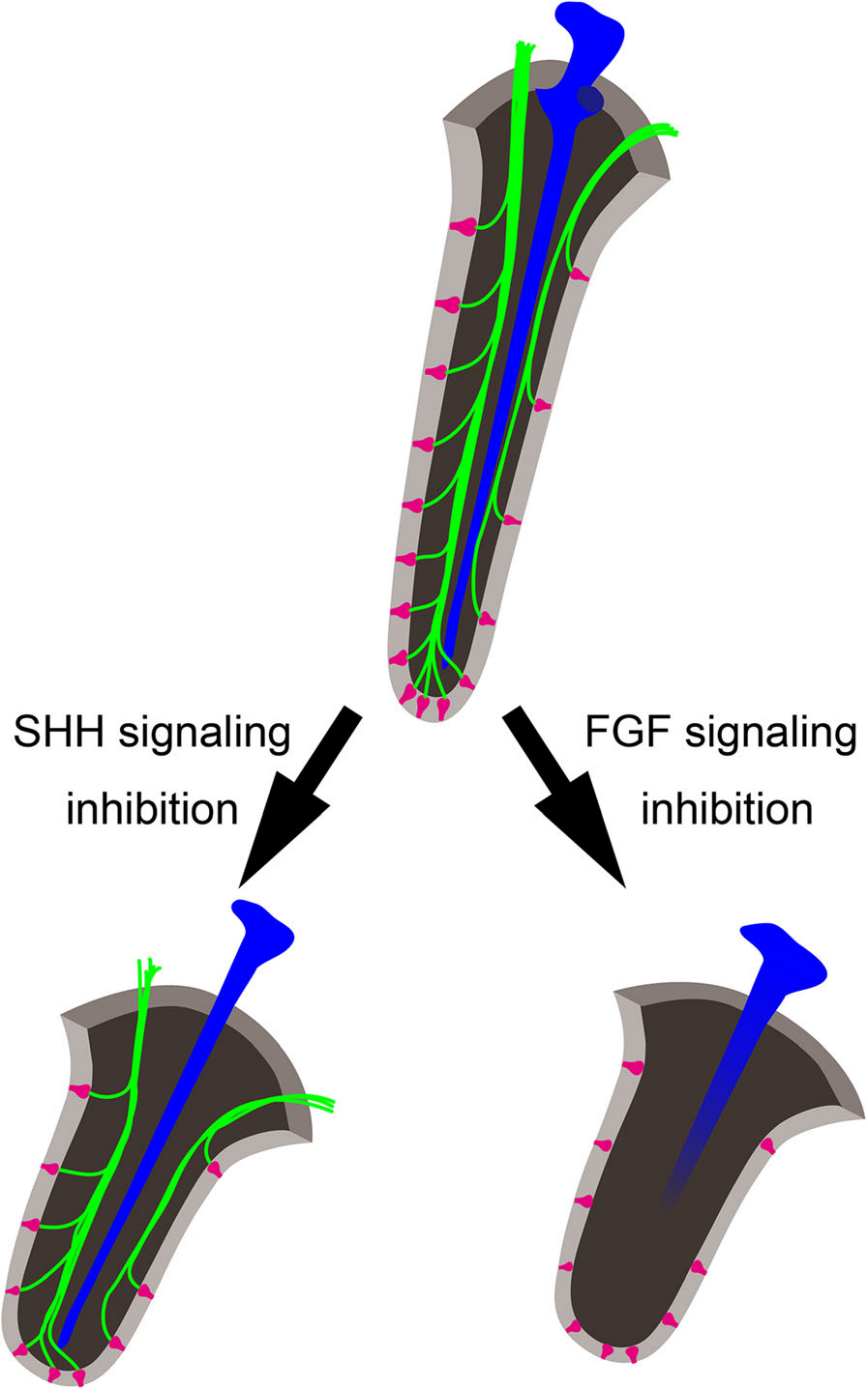
Summary of effects of SHH and FGF signal inhibitors on barbel formation

We found that SHH signal inhibition resulted in maxillary barbel length reduction and elimination of mandibular barbels. Importantly, barbel cartilages were malformed in cyclopamine-treated embryos, with maxillary barbel cartilages narrow and short, although they reached the distal end of the reduced barbel (Fig. 10). Furthermore, mandibular barbel cartilages disappeared with cyclopamine treatment. These results suggest that reduction in barbel length in cyclopamine-treated embryos may be due to the malformation of cartilages.

In contrast to the cartilaginous tissues, peripheral nerves of the maxillary barbel appeared unaffected by cyclopamine treatment; the maxillary (mx) and mandibular (md) rami of the trigeminal and facial nerves extended to the distal end of the maxillary barbel, consistent with controls (Fig. 10). It is important to note that in sea catfish, endings of the mx and md represent a specific “loop-shaped” morphology at the basal side of taste buds [7]. Previous work has also shown that this specific innervation pattern is already formed at the embryonic stage. Our study showed that in cyclopamine-treated embryos, these mx/md endings were present at the basal sides of taste buds on barbels. Thus, the maxillary barbel ramus of trigeminal and facial nerves may be able to contact taste buds successfully in the cyclopamine-treated embryos as they did in the control group. However, unlike in the control specimens, a large number of small branches of peripheral nerves could be observed in cyclopamine-treated embryos. This abnormal nerve morphology may be due to the defasciculation of the axonal bundle. If this is the case, SHH may be involved in the fasciculation of maxillary nerve axons.

Taken together, these data suggest that SHH signaling may contribute to the formation of catfish craniofacial skeletal elements, including barbel cartilages.

### Role of FGFs in barbel development

Amur catfish embryos treated with SU5402 showed reduced lengths of maxillary and mandibular barbels. However, unlike in cyclopamine-treated embryos, their maxillary size seemed unaffected. Thus, FGF signaling may specifically contribute to the formation of both maxillary and mandibular barbels, at least under the present experimental conditions (Fig. 10). Embryonic expression of *SaFgf8* mRNA was detected in maxillary barbel buds; thus, *SaFgf8* may be involved in the establishment of the anteroposterior and dorsoventral axes of the maxillary barbel. However, *SaFgf8* transcripts appeared absent in the mandibular barbel primordia. Because SU5402 is an FGFR-specific tyrosine kinase inhibitor, it may be that other *Fgf* family molecules are expressed in the developing mandibular barbel. If so, maxillary and mandibular catfish barbels are formed via independent molecular mechanisms. Thus, in future studies, it will be necessary to observe the expression pattern of other *Fgf* members in mandibular barbel primordia. Importantly, in contrast to cyclopamine treatment, development of peripheral nerves was severely affected by SU5402 treatment. This strongly indicates that the FGF signal is involved in the outgrowth of the peripheral nerves into the craniofacial region of Amur catfish (Fig. 10). It was previously reported that FGF8 regulates axon guidance factors, semaphorins, in rat embryos [57]. It may thus also be the case that FGFs regulate semaphorin expression in catfish. Moreover, although taste buds were observed on barbels, we could not identify either peripheral nerves axons or “loop-shaped” forms on the basal side of barbel taste buds. Thus, it is likely that peripheral nerves did not appropriately innervate the taste buds, despite the presence of taste buds on the barbel. Previous studies have demonstrated that development of taste buds is independent of neuronal innervation in amphibians [58–61]. Catfish taste buds may thus be able to differentiate independently without interaction with nerves (Fig. 10). We also found that maxillary and mandibular barbel cartilages were affected by SU5402 treatment. Of those, maxillary barbel cartilage did not elongate to the distal end of the barbel, and furthermore, mandibular barbel cartilages could not form. These results indicate that FGF signaling is involved in the patterning of neuronal and cartilaginous elements in catfish barbels.

### Contribution of SHH and FGF signaling to development of body surface taste buds

One defining characteristic of catfish is that they have taste buds distributed across the entire body surface. Our study showed that cyclopamine-treated embryos had decreased numbers of taste buds compared with control embryos, although their spatial distribution appeared largely normal. Conversely, taste buds in the mandibular region appeared to increase in cyclopamine-treated embryos. This may be due to the severely defected mandibular and barbel characteristics, such that we were unable to distinguish between barbels and the mandibular region. These results suggest that SHH signaling regulates the number of taste buds on the body surface. This is partially consistent with a report on cavefish, a member of Siluriformean Otophysi, in which cyclopamine treatment led to abnormal numbers of taste buds [2, 62]. Thus, SHH-dependent taste bud morphogenesis on the lip may be a synapomorphy in Otophysi. Importantly, it has been reported that SHH signaling regulates taste bud formation via a Wnt/β-catenin signaling pathway in mice [34, 35, 37, 38]. Therefore, in future studies, it will be important to identify whether the development of catfish taste bud distribution over the body surface is mediated by Wnt/β-catenin signaling.

In contrast, ectoderm-derived taste buds distributed over the body surface were reduced in SU5402-treated embryos. In the zebrafish pharyngeal region, the FGF signal derived from the endoderm is required for the formation of taste buds (taste receptor cells) and miR-200 family and Delta-Notch signaling play key roles in this process [39]. Thus, in the catfish, it may be that FGFs promote taste bud formation in ectoderm-derived tissue in addition to the endoderm.

Cumulatively, both FGFs and SHH signals may be involved in the formation of taste buds in the catfish. Since FGFs and SHH are paracrine signaling molecules, receptors for these factors may be expressed on the cell surface of taste buds to transduce their signals. Given that taste bud shapes are diverse not only between species but also within individuals, depending on their locations on the body [63], it is possible that this diversity in shape and form derives from different contributions of these morphogenesis-signaling factors.

## Conclusion

Catfish have unique developmental processes for forming expanded jaws, barbels, and taste buds over the body surface. These elements provide catfish-specific characteristics that are constructed following novel developmental programs. In the present study, we found that SHH and FGF signaling is involved in the formation of catfish-specific morphology. Because these molecules are present in all vertebrates and play a key role in various developmental stages, spatial or temporal changes to their expressions based on modification of the upstream regulatory genes may lead to several unique elements, as an evolutionary novelty, in Siluriformes. Notably, these molecules may act as important tools for the diversification of teleosts. Further study of Siluriformes will provide greater insight into this field of evolutionary and comparative biology.

## Additional file

Additional file 1: **Figure S1.** Phylogenetic tree of *Fgf8* and *Shh* in vertebrates. *SaFgf8* and *SaShh* are indicated by red boxes. we could not identify whether *SaShh* is belong to *Shha* or *Shhb* clusters. This may be due to the partial sequence of *SaShh* obtained in this study. (PDF 155 kb)
