# Supplementary figures and images for: FGF- and SHH-based molecular signals regulate barbel and craniofacial development in catfish
Source: Zoological Lett. 2019 Jun 14;5:19. doi: 10.1186/s40851-019-0135-1 (PMC6570838; doi:10.1186/s40851-019-0135-1)

## Fgf8

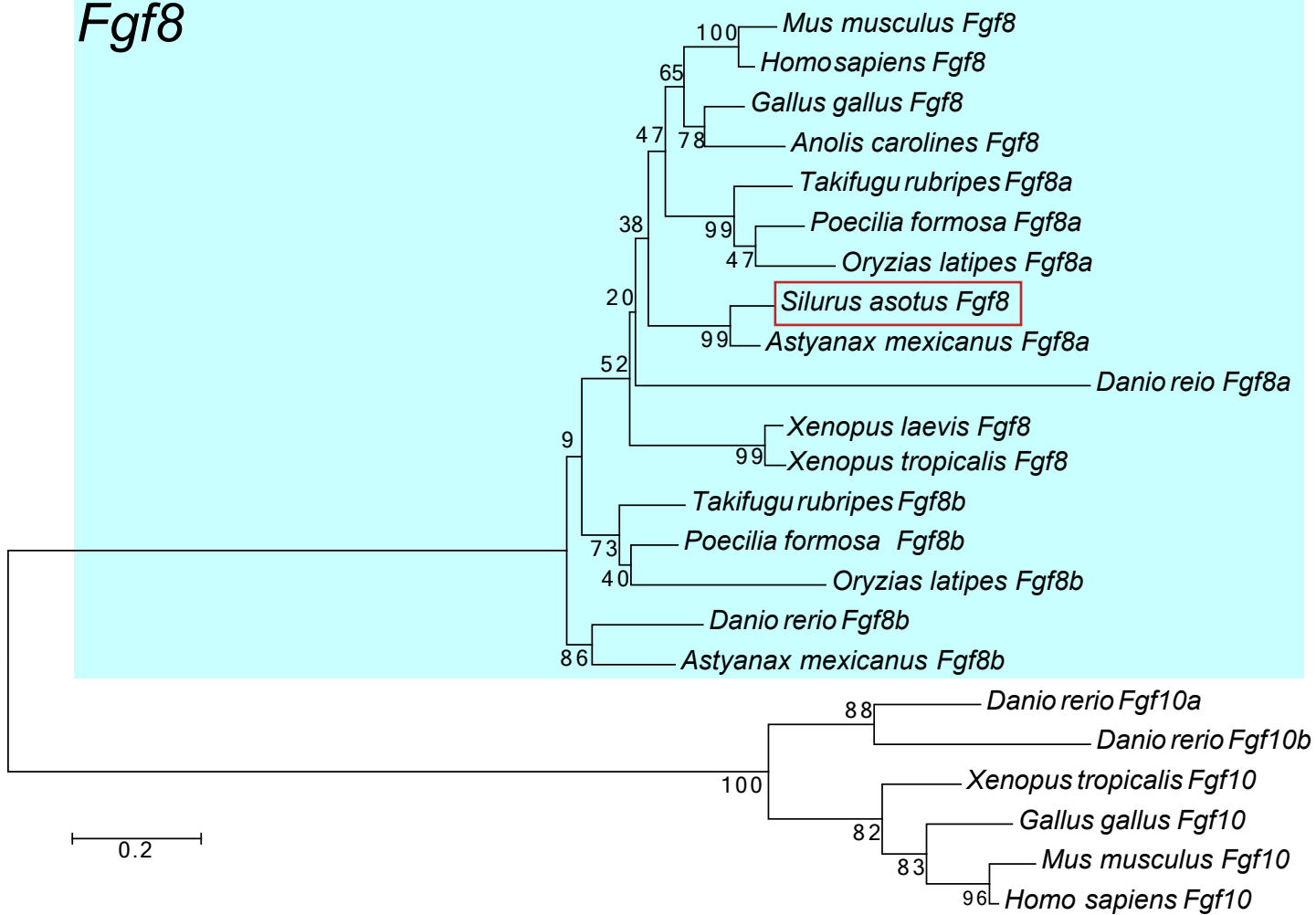

## Shh

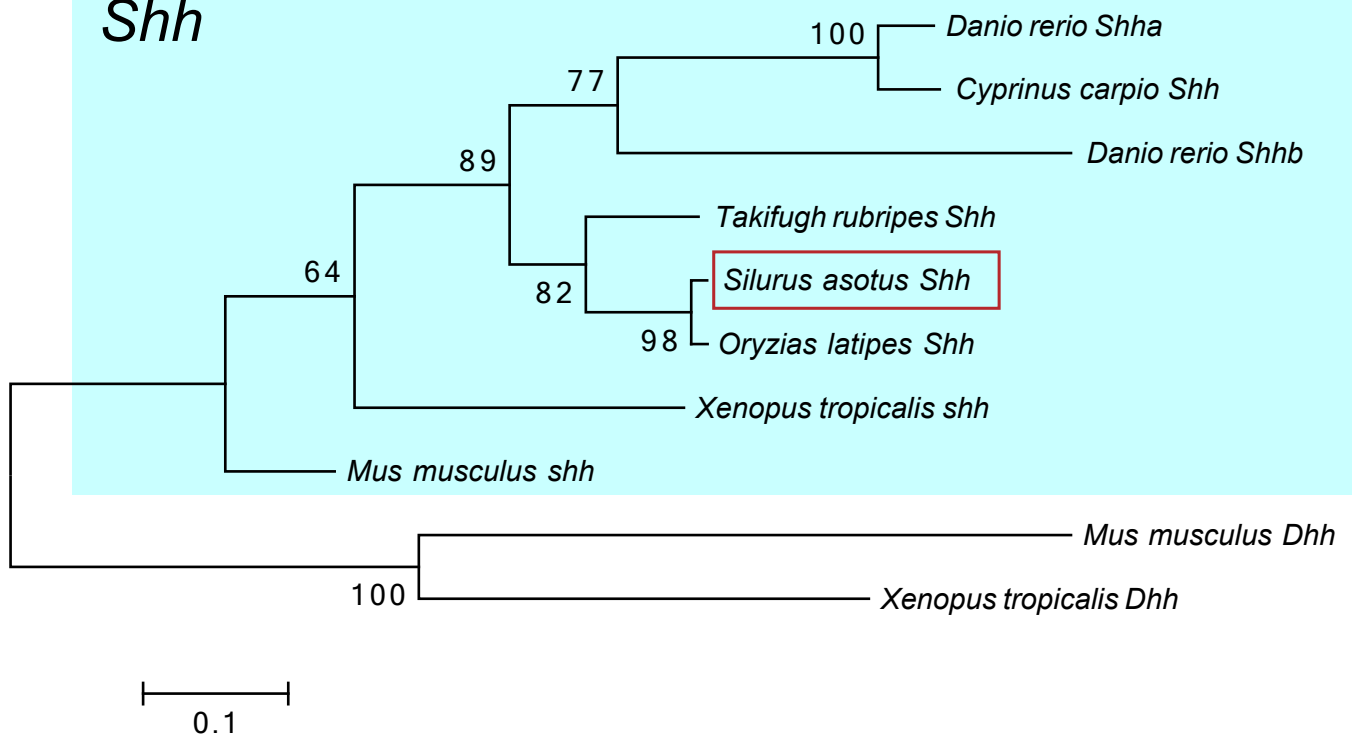

Supplement: Supplementary file 1 — Figure S1. Phylogenetic tree of Fgf8 and Shh in vertebrates. SaFgf8 and SaShh are indicated by red boxes. we could not identify whether SaShh is belong to Shha or Shhb clusters. This may be due to the partial sequence of SaShh obtained in this study. (PDF 155 kb) [file 40851_2019_135_MOESM1_ESM.pdf]
